# Inertial Sensor-Based Robust Gait Analysis in Non-Hospital Settings for Neurological Disorders

**DOI:** 10.3390/s17040825

**Published:** 2017-04-11

**Authors:** Can Tunca, Nezihe Pehlivan, Nağme Ak, Bert Arnrich, Gülüstü Salur, Cem Ersoy

**Affiliations:** 1 Department of Computer Engineering, Computer Networks Research Laboratory (NETLAB), Bogazici University, 34342 Istanbul, Turkey; nezihe.pehlivan@boun.edu.tr (N.P.); bert.arnrich@boun.edu.tr (B.A.); ersoy@boun.edu.tr (C.E.); 2 65+ Elder Rights Association, 34337 Istanbul, Turkey; nagme@yaslihaklaridernegi.org (N.A.); gulustu@yaslihaklaridernegi.org (G.S.)

**Keywords:** gait analysis, wearable sensors, inertial sensors, spatio-temporal gait metrics, Kalman filter, neurological disorders, Parkinson’s disease

## Abstract

The gold standards for gait analysis are instrumented walkways and marker-based motion capture systems, which require costly infrastructure and are only available in hospitals and specialized gait clinics. Even though the completeness and the accuracy of these systems are unquestionable, a mobile and pervasive gait analysis alternative suitable for non-hospital settings is a clinical necessity. Using inertial sensors for gait analysis has been well explored in the literature with promising results. However, the majority of the existing work does not consider realistic conditions where data collection and sensor placement imperfections are imminent. Moreover, some of the underlying assumptions of the existing work are not compatible with pathological gait, decreasing the accuracy. To overcome these challenges, we propose a foot-mounted inertial sensor-based gait analysis system that extends the well-established zero-velocity update and Kalman filtering methodology. Our system copes with various cases of data collection difficulties and relaxes some of the assumptions invalid for pathological gait (e.g., the assumption of observing a heel strike during a gait cycle). The system is able to extract a rich set of standard gait metrics, including stride length, cadence, cycle time, stance time, swing time, stance ratio, speed, maximum/minimum clearance and turning rate. We validated the spatio-temporal accuracy of the proposed system by comparing the stride length and swing time output with an IR depth-camera-based reference system on a dataset comprised of 22 subjects. Furthermore, to highlight the clinical applicability of the system, we present a clinical discussion of the extracted metrics on a disjoint dataset of 17 subjects with various neurological conditions.

## 1. Introduction

Gait abnormalities are crucial indicators of the state and the progression of disorders that have motor symptoms. Therefore, gait analysis is essential for clinicians both for diagnostic and treatment purposes, as the medication effects and the disease progression can be monitored through gait-related symptoms. Gait analysis is especially important for neurological diseases, which require constant monitoring and treatment adjustments. Unlike temporary/treatable or static orthopedic diseases, some neurological diseases are associated with disabling, progressive gait disorders and increased risk of falls for the remainder of the patient’s life. These patients show fluctuations and variable response to treatment options. Treatment needs to be tailored to their changing needs in the real-life settings. Many neurological conditions are life-long, and managing the gait-related symptoms can have a significant role in increasing the quality of life.

The occurrence and severity of gait abnormalities vary significantly from patient to patient due to possibly multiple underlying problems that are hard to detect solely by clinical observation. Hence, any additional system that can estimate gait metrics is beneficial to aid and help the clinicians confirm their observations and diagnoses. Moreover, gait assessment is essential in predicting and preventing falls, which can result in significant morbidity and mortality. Being able to quantify the treatment effects is also an important aspect of clinical efforts, which must be adapted to operate on limited observation and data, since outpatient visits are usually infrequent and short. Moreover, some metrics, such as feet stance durations, which are on the order of tenths of a second, might be hard to assess by only visual inspection.

Camera-based 3D motion capture systems and instrumented walkways are considered as the gold standard in gait analysis in terms of accuracy. However, both of these systems are suitable for only hospitals or hospital-like settings, such as specialized gait analysis clinics, due to their size, high cost and the need for trained professionals to operate them. These characteristics render these systems rare and inaccessible to a substantial portion of the patients. Therefore, pervasive and objective gait assessment systems that can be deployed easily in non-hospital settings (such as homes or small private clinics) have a significant advantage. Such a system may also complement the gold standard, as a more frequent and accessible counterpart.

The recent developments in inertial sensors made them promising candidates for monitoring motor symptoms and assessing gait dysfunctions. The decreases in their costs and sizes have made them more pervasive and attractive than ever. Attachable to almost any body part via straps, they have become viable, non-obtrusive alternatives for analyzing gait. An inertial measurement unit (IMU) consists of multiple inertial sensors (namely an accelerometer, a gyroscope and a magnetometer) combined into a single device. Enhanced with wireless communication capabilities, multiple IMUs can gather real-time, synchronized data from multiple parts of the body that are active in gait. The recent proposals of body sensor network (BSN) architectures provide convenient middleware frameworks [1] that greatly alleviate the challenges in multi-sensor fusion [2]. However, processing the data to estimate the 3D position evolution of the attached limb is still challenging due to the error characteristics of the underlying sensors, which are explained in detail in Section 2.2.1.

Using IMUs for the purposes of gait analysis also attracts the attention of the academic community, as there are numerous studies in the literature that employ IMUs [3,4]. Especially for lower-body gait analysis with foot-located IMUs (either via straps or insoles), an accurate methodology that combines zero-velocity updates combined with Kalman filtering has been established and well tested [5,6]. The ultimate aim of a gait analysis system is to extract a variety of standardized gait metrics to be easily interpreted by a clinician. However, a system solely consisting of wearable sensors, without the aid of infrastructural system elements, rarely achieves completeness in terms of gait metrics. The literature still lacks a complete system that can be easily used by non-professionals in a non-hospital setting.

As a step towards this goal, in this study, we present an infrastructure-less system based on the above-mentioned established methods that is extended with a novel methodology to improve the operation of the system in non-hospital settings where data collection and sensor placement errors are possible. Specifically, we propose a medial-lateral (ML) foot angular change detection method that is able to operate regardless of the sensor placement on the foot, which is in turn used in an initial-contact (IC) and foot-off (FO) events detection algorithm that is adaptive to pathological gait. Our system has minimal requirements on the data collection procedures and setup and is able to extract a rich set of spatio-temporal gait metrics that also include metrics about turning steps, which are disregarded in many existing studies. The contributions and highlights of the proposed system can be summarized as follows:

- The proposed system is completely mobile, requiring only two IMUs and a data-processing notebook, and is usable in non-hospital settings, without the need for infrastructure.
- We propose a robust initial contact/foot-off (IC/FO) detection method that is able to operate under imperfect foot-relative sensor placement and orientation conditions. This method is also adaptive to pathological gait (i.e., foot dragging, the absence of the heel strike or left-right asymmetries), where the typical temporal phases of gait may not be observed.
- The system is able to detect and operate with side, back and turning steps, in contrast to solely walking straight, thanks to a novel particle filter-based foot orientation estimation algorithm.
- A rich set of standard spatio-temporal gait metrics is extracted, which can easily be interpreted by a clinician without the need for professional support. The extracted metrics include stride length, cadence, cycle time, stance time, swing time, stance ratio, speed, maximum/minimum clearance and turning rate.
- In addition to numeric metrics, a new lateral stride profile visualization method is proposed, to give instant insight on spatial gait characteristics.

We conducted a validation study on 22 subjects, using a depth camera (Microsoft Kinect v2) as a reference system. Furthermore, we collected data from 17 patients with various neurological conditions and discussed the extracted gait metrics and visualizations to show how the output of the system can be used for clinical purposes.

The paper is organized as follows: In Section 2, we present some background information on the human gait characteristics, standard spatio-temporal gait metrics, IMU error characteristics and data collection challenges. In Section 3, we discuss the related work on gait analysis with IMUs that have practical relevance to our study. In Section 4, we present the details of the system setup and the data collection procedures. In Section 5, we briefly explain the state of the art zero-velocity update and Kalman filtering techniques, and we delve into the details of the proposed new methods. In Section 6, we present the results of the validation study and the experimental study on real patients, focusing on the clinically important findings. The paper is finally concluded in Section 7, with final remarks and future research directions.

## 2. Background

In this section, we provide the readership with a brief overview of human gait characteristics and the standard gait metrics that are crucial for detecting different factors leading to pathological gait. Moreover, we discuss the challenges of working with wearable IMUs with the aim of accurate gait analysis, both in terms of the error characteristics of the underlying hardware and the difficulties in deployment and data collection. These challenges serve as the rationale behind the aims of this study, explicitly, to come up with a robust gait analysis solution that is minimally affected by the typical errors associated with IMUs, either arising from the human factor or the hardware.

### 2.1. Human Gait Cycle and Gait Metrics

**Figure 1:**
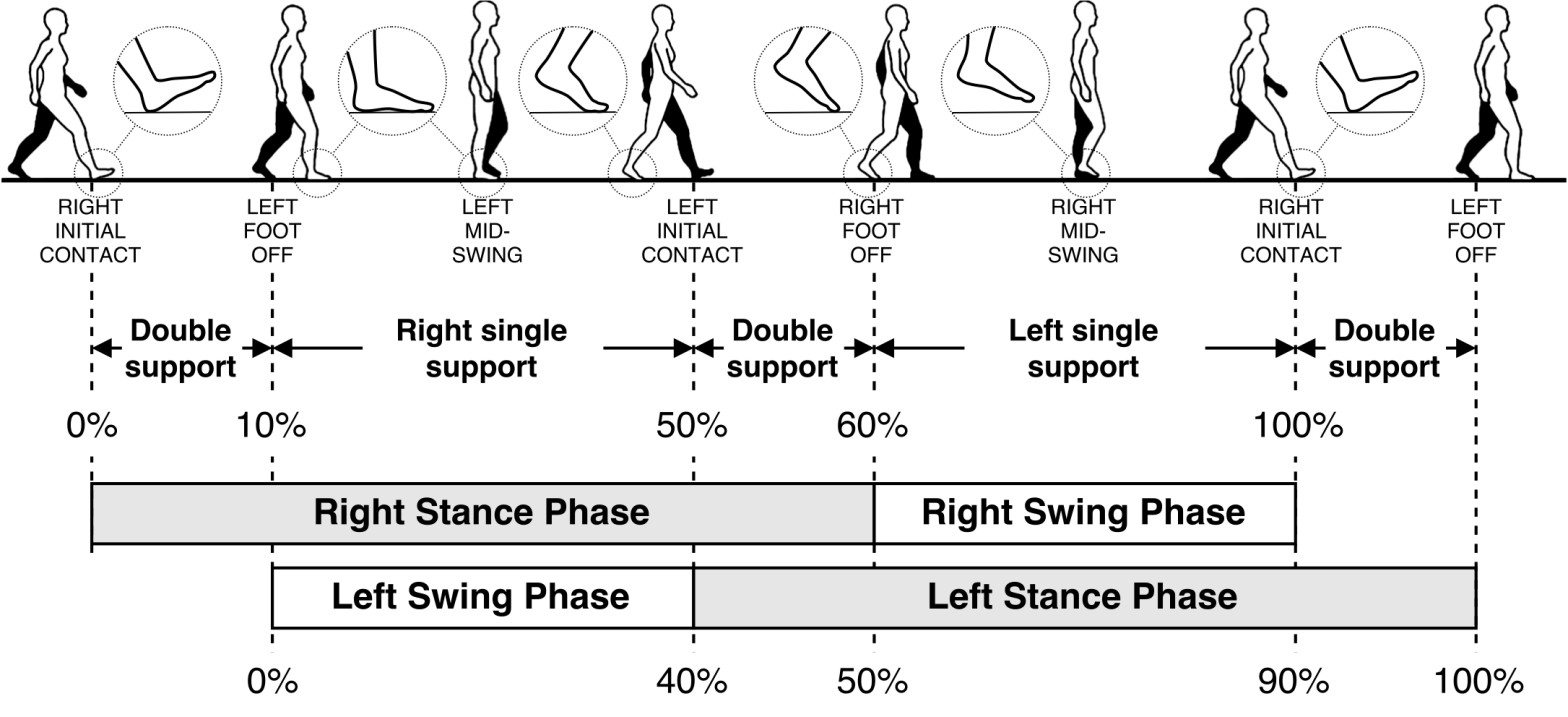
Human gait cycle.

A single gait cycle is defined as the period between any two successive repetitive gait events [7]. The primary events and phases in the gait cycle are presented in Figure 1. It consists of two major phases, namely stance and swing phases. The stance phase begins with initial contact (IC), which marks the beginning of the load transfer to the ground-contacting foot. The stance phase ends with foot-off (FO), which marks the complete lifting of the load. Swing phase starts with FO and ends with IC, which is the phase where the actual foot motion occurs. Normally, the stance phase constitutes approximately 60% of the whole gait cycle, and the swing phase constitutes the remaining 40% [7].

Closeups of the gait events of the right foot are also presented as zoom-in circles in Figure 1. The IC and FO events are alternatively named as heel-strike and toe-off, respectively. However, these alternative terms are only valid for normal gait. In pathological gait, the first contacting point of the foot may not be the heel (especially in Parkinsonian shuffling gait, where the foot strikes the ground all at once). Similarly, toe may not be the last lifted part of the foot in some rare cases. Hence, the terms IC and FO are more accurate. Moreover, there may be cases where the subject’s feet do not leave the ground at all (i.e., foot dragging). In this case, the FO event is defined as the instant when the forward motion starts, and opposingly, the IC event is when the forward motion stops.

An insightful case of pathological gait is observed in Parkinson’s disease (PD). Even though this study is not solely focused on PD, many of our patients suffer from it. Moreover, PD shares many gait symptoms with other neurological disorders, rendering it important for understanding common gait abnormalities [8]. Typical gait in PD is shuffling gait, historically so-called “marche a petits pas”, with slow and small steps [9]. PD usually starts on one side of the body, resulting in asymmetry. As the disease progresses, the feet may become magnetic, hard to lift off the floor, and in some cases, there is freezing of gait. Comorbidities like vascular brain diseases may result in additional gait problems due to paresis and imbalance. Sensory problems like poor eye sight and polyneuropathies that interfere with sensation may also disrupt balance [10]. Conditions like spinal stenosis or normal pressure hydrocephalus may cause problems confined to the lower extremities [8]. It is important to detect different components of gait problems when making a diagnosis and following the patient.

Extracting a variety of spatio-temporal gait metrics help us isolate and identify these components. Standard spatio-temporal gait metrics that have clinical significance are listed in Table 1. In this study, we are able to estimate all, but two of these metrics: step length and walking base. Estimating step length may appear trivial at first glance; however, the definition presented in Table 1 requires the spatial synchronization between two feet, i.e., knowledge of relative displacement and orientation between two feet at all times. The proposed system tracks the position change of the feet separately with respect to their initial positions; thus, a positional drift is imminent. Hence, the current state of our system does not guarantee spatial synchronization. Walking base also requires spatial synchronization. There are proposals in the literature that employ additional equipment to achieve this synchronization (such as a small camera mounted on a foot to detect the other foot when they are adjacent [5]); however, we chose not to employ additional sensors in this study since they would possibly hinder the non-obtrusiveness of the system.

### 2.2. Challenges of Working with IMUs

Even though the evolution of technology has improved the underlying sensors in a typical IMU over recent years, there are still challenges that need to be faced. Some of these challenges can be overcome by the state of the art proposals in the literature, while some of them are not yet addressed or solved. Moreover, some of the solutions in the literature assume controlled data collection environments (error-less system setup and deployment) and perfectly executed data collection procedures, which are rarely achievable even in a professional-aided clinical setting. This issue gets even more challenging in non-hospital settings, which we ultimately target in this study.

#### 2.2.1. Error Sources in IMUs

IMUs consist of accelerometers, gyroscopes and magnetometers. In theory, acceleration measured by the accelerometer can be integrated twice to acquire the relative change in position. Similarly, the angular velocity measured by a gyroscope can be integrated to compute the relative orientation (attitude) change. However, in practice, these sensors are susceptible to noise and other small errors, which cause significant drift when the raw measurements are integrated. It should be noted that accelerometers or inclinometers could also be used to directly determine the orientation (more accurately, the direction of gravity relative to the device) without the need for integration; however, external accelerations applied to these sensors may confuse the detection of the gravity vector. Hence, when determining 3D orientation, usually data from multiple sensors are fused.

**Figure 2:**
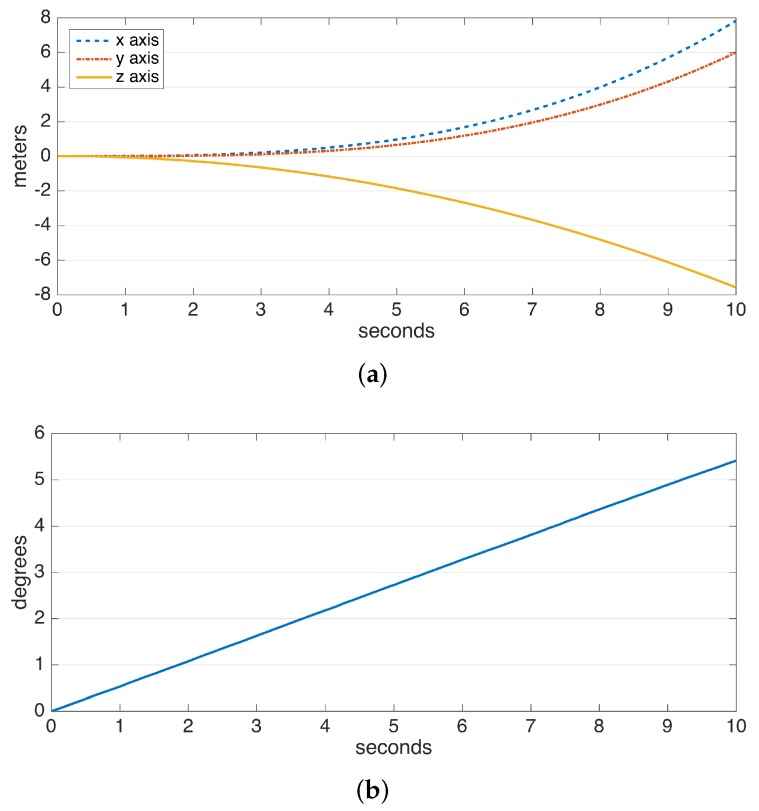
Typical drifts observed in the integration of data from the (**a**) accelerometer and (**b**) gyroscope.

The drift errors due to integration are demonstrated in Figure 2. The data are collected from a perfectly stationary IMU. The gyroscope and accelerometer drift result in a linearly-growing error in angle and a position error growing proportional to $t^{2}$, respectively. Especially the position error, which rises to the order of meters in a few seconds and to the order of hundreds of meters in a minute, makes it practically impossible to use solely double integration for position estimation. Hence, there is a need for additional domain knowledge to estimate and eliminate drift periodically. Fortunately, human steps are of a periodic and frequent nature.

The state of the art to cope with the accelerometer and gyroscope drifts involves the combination of a technique referred to as the zero-velocity update, which exploits the periodic nature of the human gait, and a Kalman filter to track and correct the correlations of the state errors [5,6]. These techniques are also encountered in the pedestrian navigation literature, where high position accuracy is the primary concern. Brief explanations of zero-velocity updates and Kalman filtering are provided in Section 5.1 and Section 5.2, respectively. In this study, we also employ these techniques, but we support them with novel additional methodology to establish a robust system that is minimally affected by unintentional faults in deployment and data collection, such as sensor placement imperfections, loose time synchronization between multiple sensors and errors regarding the application of data collection procedures (e.g., insufficient calibration period). In the next section, these typical errors are investigated in detail. Our methods also consider pathological gait and do not rely on assumptions of normal gait.

#### 2.2.2. Operational Challenges and Difficulties in Data Collection

Typical sensor placement locations for lower-body gait analysis include feet, shanks, thighs and trunk; each with distinct advantages and disadvantages. A complete system with multiple sensors in each of these locations may provide the most information, but with each sensor, the obtrusiveness increases. Considering the objective of extracting as many spatio-temporal gait metrics as possible without diminishing mobility and convenience, placing only two sensors on the feet stand out as a viable tradeoff between obtrusiveness and completeness, since it allows us to employ zero-velocity updates to achieve high spatial accuracy, which in turn can be converted into a rich set of gait metrics.

The IMUs may typically be collocated with the feet via straps or insoles. Insoles offer a more stable sensor placement, but their costs are typically higher. It goes even further up with the need to have different sizes readily available for different shoe sizes. Moreover, insoles are only usable with certain types of shoes, and they are not suitable for shoeless (bare-foot or with socks) usage. On the other hand, length-adjustable straps (typically Velcro) do not have these restrictions at the expense of decreased sensor stability. Because of this and the unrestricted deployment options, defining and enforcing a standard way of strapping the sensor on the foot is impossible; the specific location and the orientation of the sensor may change from session to session and patient to patient. Therefore, assuming a specific sensor orientation relative to the foot (e.g., a specific IMU axis aligned with either the medial-lateral or the anterior-posterior) is highly unrealistic. It should also be noted that the controlled environments where the validation experiments are conducted are not existent in the field. Moreover, a successful system should be easily usable by a non-professional, who may make mistakes during a data collection procedure. Such mistakes should not render valuable data unusable.

Human errors may sometimes be even inevitable, especially in time-constrained cases where the time allocated for data collection is limited and repetitions to remedy errors are not possible. Clinicians can spend limited time for each patient, which may simply prevent perfect data collection. The same also goes for the patients, who may not want to spend hours until the perfect data are finally achieved. In short, we must make use of the data we get, no matter the errors.

Other data collection errors include the initiation of the sensors after the subject starts walking. Many existing studies rely on a calibration period where the subject is expected to perform a specific movement pattern (either standing still or walking on a straight line), for correct operation afterwards. Such a period may not be available due to either human error (e.g., the data collecting personnel may forget about the calibration period altogether, or the subject starts walking immediately without waiting for a cue), or hardware problems leading to data loss (e.g., wireless communication issues at the start of a session that render calibration data unusable). These examples are from the very problems that our research group encountered in the recent few years. Therefore, the methods of this study are designed to operate without the need for a calibration procedure.

Hardware problems may also lead to time-synchronization problems between multiple IMUs. Most IMUs on the market use timestamps to label the data; but this rarely alleviates the problem, since usually in gait applications, a synchronization on the order of milliseconds is required, which is very difficult to achieve even from the beginning. The internal clocks of the sensors may easily fall out of sync due to battery issues, and a perfect sync rarely lasts a few days. Our methodology does not rely on time-synchronization, as different feet are tracked separately, and the metrics are aggregated in the metric extraction phase rather than in raw data analysis. Battery life is also an important concern for systems targeting continuous gait monitoring. Although efficient power-aware motor activity-monitoring BSN architectures have been proposed [11], it still remains a challenge. Of course, these hardware problems are mitigated each day by the technological improvements and may no longer be of question in the future. However, human errors will certainly remain to be a reality, and unfortunately, some of these errors are simply unavoidable. As also dissected in the next section, only a small portion of the related literature stresses this reality. One of the primary aims of this study is to fill this gap by proposing a robust gait analysis system that is minimally affected by such errors and makes minimal assumptions on the correct application of the data collection procedure.

## 3. Related Work on Gait Analysis with IMUs

In this section, we present the overview of the studies that have some relevance to our study, not only in terms of sensor placement, but also in terms of the employed methodology. We highlight the advantages and drawbacks of these methods with respect to the current state of our system. The details of the overviewed studies are presented in Table 2. The aims of the studies along with the corresponding methodology (highlighted in parentheses), the number and placement of the sensors, the number of test subjects and the strengths and weaknesses of the studies are included.

Estimating the temporal metrics requires the detection of IC and FO events, which separate the stance and swing phases. A widely-adopted method for IC/FO detection is peak detection on the raw acceleration or gyroscope (angular velocity) signals. IMUs placed on either the top of the feet [6,15,22,25] or on the ankles/shanks [16,17] are used for this purpose. Peak detection can be further complemented with additional features, such as the derivative of the acceleration signal and fed into a hidden Markov model (HMM) to detect the underlying states of a stride more accurately [18]. Another alternative placement is the trunk at the expense of decreased accuracy, which can be improved with the more advanced methodology of wavelet-based time and frequency analysis [27]. These methods achieve a reasonable timing accuracy; however, the majority of the studies make the assumption of normal gait and foot fall. The peaks required to detect IC events are only observed if there is a pronounced heel strike. Only a few studies consider the possibility of pathological gait (even foot fall) [15]. The IC/FO detection method proposed in our study aims to produce accurate results even in this case.

Some studies employ force sensors integrated with a shoe [21] or an insole [23] to increase the timing accuracy and address the challenges of pathological gait. Even though these approaches produce superior detection performance at the expense of additional hardware, they are more costly compared to the only-IMU solutions and have decreased convenience for the patients due to the need to wear specific shoes and insoles (which should be made available in different sizes for different patients, also contributing to the increased cost).

Extracting spatial metrics such as stride length are a more challenging problem due to the technical challenges associated with IMUs. A well-explored method for stride length estimation is to construct a geometric human locomotion model using the joint angles estimated via the IMUs [14,16,19,20]. The absolute 3D-orientation of the IMUs can be accurately computed via the fusion of acceleration and gyroscope data, which can then be converted into joint angles using the orientation differences between adjacent sensors [12]. This approach also enables the virtual reconstruction of the skeletal motion of the lower body. However, the spatial accuracy of this method is generally low, since the orientation errors even as low as a few degrees correspond to several centimeters in the constructed geometric model, and the errors in adjacent joints produce a cascading effect when determining the displacement of the feet. Another drawback of this method is the need to measure the exact lengths of the lower body segments of the subjects, which adds to the complexity of the pre-test preparations.

Another approach to determine spatial metrics is double integration of the acceleration signal to measure the displacement [13,17,21]. To cope with the drift errors accumulating due to integration (discussed in Section 2.2.1), the integration window should be reset periodically. Even small errors in the reset timing lead to large displacement errors. A viable approach to determine the reset times is to detect feet zero-velocity phases [22,28], which is only applicable for foot-placed IMUs. However, zero-velocity updates only prevent the drift errors from growing beyond acceptable levels and do not attempt to minimize the already accumulated error. A better approach is to use Kalman filtering aided with zero-velocity updates to track the errors and correct them retrospectively [5,6], which leads to high spatial accuracy. Another method to determine the stride length is to use a single trunk-placed sensor and utilize double integration along the vertical gravity-aligned vector, which is then converted into horizontal displacement using the inverted pendulum model [27]. The advantage of this method is using only a single IMU; however, the spatial accuracy of this approach is low.

Other studies of interest include [24,26,29], which are quite different compared to the other studies reviewed in this section, as they do not aim to extract standard gait metrics. However, they are worthy of attention since they aim to extract balance and symmetry-related upper-body features from the raw data of sensors placed on the trunk. Currently, we do not employ these approaches, but they are important contributions that may extend the current capabilities of our system in the future, at the expense of an additional sensor.

For further reading, surveys on gait analysis with IMUs include [3,4]. The existing literature suggests that even though extraction of spatio-temporal gait metrics is in the focus of many studies, a complete system that uses an efficient methodology such as zero-velocity update-aided Kalman filters-smoothers and extracts a wide range of spatio-temporal metrics (including spatial metrics in the elevation domain) is still not present. Moreover, the majority of the proposals are suitable for normal gait patterns, and their applicability to pathological gait is questionable. It should be noted that this is not due to the targeted standard gait metrics, as they are able to capture many gait abnormalities. For instance, a patient with advanced PD may have magnetic feet, which would increase the stance times (due to the difficulty to lift the feet off the ground). This would lead to a stance ratio higher than the typical value of 60%. A system that makes the assumption of normal gait may be able to compute a metric in case of pathological gait, but the accuracy of the computed value would be very low. Our study aims to achieve a high accuracy, even in pathological cases.

Another weakness observed in many studies is the lack of validation studies with a sufficient number of real patients. A final observation is that, even though all of the studies dissected in this section have clinical significance, the technical contributions and clinical discussion rarely come together; hence, the applicability of the output of existing systems for clinical purposes is not clear. We aim to fill this gap by providing real examples of what our system can detect, by combining them with the interpretation of a clinician.

Comparing the Kalman filtering-based methods and other methods in the literature, it is evident from the evaluation results that the former is superior in terms of accuracy. Hence, we believe that we are on the right track for selecting this approach as a basis to compute the spatial metrics. However, extraction of the spatio-temporal metrics is a whole different problem that requires an additional methodology. Therefore, we aim to extend the capabilities of the existing methodology, by estimating a substantial portion of the core spatio-temporal metrics presented in Section 2.1.

## 4. System Setup and Data Collection

In this section, we present the details of the core IMU-based system hardware setup and the data collection procedures. The details of the reference system used for the system validation study are not included here since it is not a part of our core system, and they are presented in Section 6.1.

### 4.1. Wearable IMU Setup

We employ EXEL ExLs3 IMUs [30] in this study. The raw data are collected at a sampling rate of 100 Hz, with acceleration in the range of ±4 gand angular velocity in the range of 1000 degrees per second. ExLs3 IMUs have wireless communication capabilities via Bluetooth and a local storage, so the data can either be transmitted in real time to a processing unit, or can be stored on the device for later acquirement and analysis.

**Figure 3:**
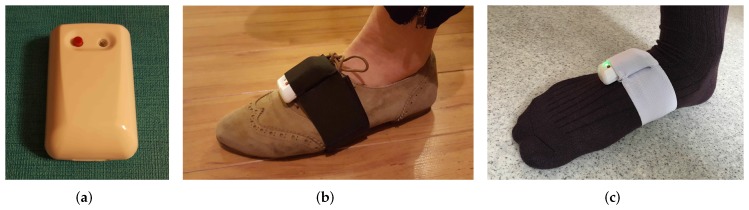
(**a**) ExLs3 IMU, (**b**) strapped to the shoe and (**c**) strapped on the socks.

The IMUs are strapped on both feet via adjustable tailor-made Velcro straps with IMU housing pockets. The ExLs3 IMU and the foot strap are shown in Figure 3. The straps are usable with shoes, socks or even bare-foot. We chose straps over insoles due to their lower cost, high convenience and ease of deployment, at the expense of decreased stability, as explained in detail in Section 2.2.2. We aim to make up for the stability issues with the novel methodology presented in Section 5.

### 4.2. Data Collection

The data collection procedure of our system has less strict requirements compared to the related work, in accordance with our objectives. Each walking session should have a sufficient number of steps (i.e., more than 20 steps), for the metrics aggregated over multiple strides to be meaningful. Naturally, longer data would yield more conclusive analysis reports, but short data do not pose any technical challenges on the system.

The system can operate with data containing back, side and turning steps, meaning that we do not enforce a specific walking pattern on the subjects; but having absolutely no forward steps in a dataset would hinder the operation of some of the system modules. Thus, in the experiments, we typically advised the subjects to walk in straight lines with turns in between to also enable the analysis of turning steps (i.e., walking in a corridor and turning back when the ends are reached).

The system does not require any calibration period whatsoever; hence, data collection may be initiated even after the subject has started walking.

## 5. Methods

**Figure 4:**
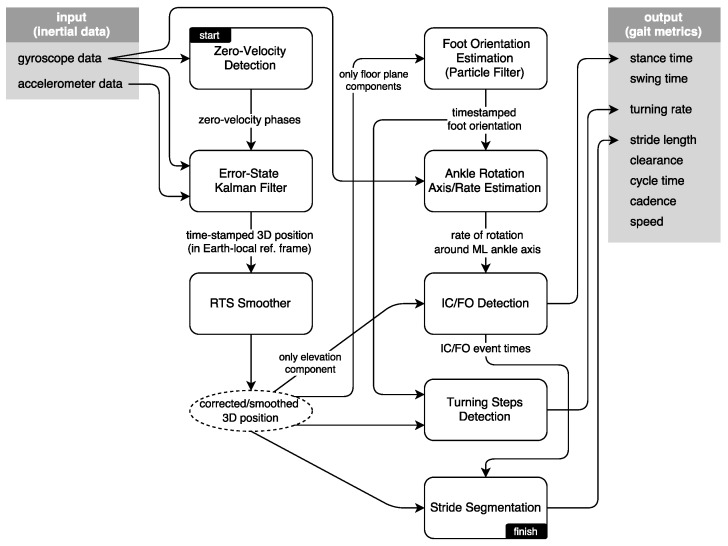
System block diagram.

The interaction of the system modules is presented in Figure 4. In this section, we present the methodology driving these modules, in an approximately sequential order. First, we present brief information on the zero-velocity updates and Kalman filtering techniques to extract the 3D position of the feet; then, we explain the smoothing approach to further mitigate the position errors during foot swing phases. Foot orientation and foot ankle rotation axis/rate estimation methods serve to mitigate the effects of sensor misplacement on the subsequent IC/FO detection algorithm, while producing additional output to be used in detecting and processing turning steps. Finally, we discuss how we extract the spatio-temporal metrics and how the output is visualized as average lateral stride profiles.

The Kalman filtering and smoothing methodology are based on the existing literature [5,6]; however, the particle filter-based foot orientation estimation method that serves to mitigate the sensor misplacement errors, the turning steps detection algorithm and the average lateral stride profile visualization method are novel. We are also able to extract metrics related to feet elevation, i.e., feet maximum clearance, which are not encountered in any of the reviewed studies. Our IC/FO detection algorithm is based on the observations in [6,31], but we extended them significantly to come up with a novel algorithm that can also operate in cases of pathological gait.

### 5.1. Zero-Velocity Updates

The state of the IMU, and coincidentally the foot it is strapped to, can be defined in terms of its attitude (3D orientation), velocity and position; $x_{t}=\left(R_{t},v_{t},s_{t}\right)$, where $R_{t}$ is the rotation matrix defining the attitude of the IMU with respect to the local Earth reference frame; $v_{t}$ and $s_{t}$ are 3-dimensional vectors specifying its velocity and displacement, respectively. In theory, assuming a known starting point and given a stream of acceleration and angular velocity readings, the relative state can be indefinitely tracked using the pedestrian navigation equations given in [32], which consist of the implementation of appropriate integration schemes. However, due to the error sources and the consequential drift as discussed in Section 2.2.1, this is not practically possible.

To cope with the position and orientation drift, a periodic correction (ground truth) of the state elements is required. Fortunately, the state vector elements are correlated with each other (e.g., velocity influences position); hence, correcting only a portion of the state improves the subsequent estimation of other elements.

Foot-mounted IMUs allow us to use the knowledge that the foot remains stationary for a short period within the stance phase. In this period (i.e., zero-velocity phase), the foot has near-zero velocity, which can be used to correct the velocity components in the state vector. Furthermore, if we assume that the walking subject does not change elevation/floors, we can also assume that the *z* component of the displacement (i.e., elevation with respect to the ground) is zero during stance phases.

**Figure 5:**
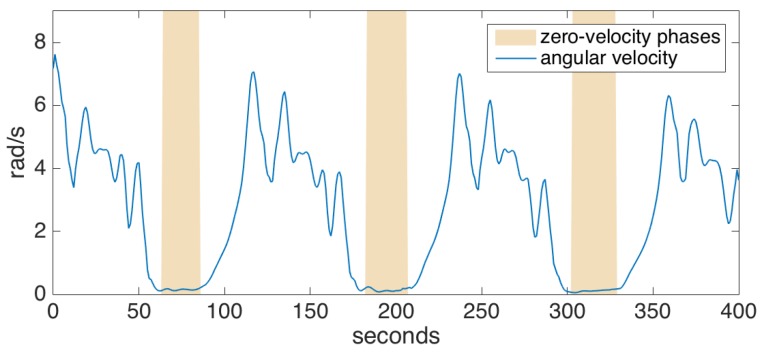
Zero-velocity phase detection.

To detect the zero-velocity phases, we check if the magnitude of the angular velocity drops below a certain threshold. To cope with noise and outliers in the data, we employ a grace period, during which the angular velocity magnitude should be under the specified threshold before we can safely assume that the foot has gone into the stance phase. An example is shown in Figure 5. Acceleration thresholding can be additionally employed in order to improve the confidence of the stance phase detector; however, we determined that the gyroscope readings are much more reliable than accelerometer readings, and such an extra mechanism barely increases the accuracy [33]. Indeed, we rarely encountered a problem with zero-velocity updates during the experiments, even for pathological gait data (e.g., the absence of heel strike or foot dragging), as also supported by the other studies utilizing the same method [5,6].

Once the zero-velocity phases are detected, we can apply the zero-vand zero-zupdates by directly modifying the corresponding state vector components. Even though this naive approach results in a significant improvement, it is not optimal, since it does not consider the correlations between the state elements. Next, we describe the better Kalman filtering approach.

### 5.2. Error-State Tracking Kalman Filter

Kalman filters are Bayesian filters that are able to accurately track correlations between the state elements with known update equations and error models.

It is possible to formulate a Kalman filter that tracks the total state of the IMU; however, non-linear update equations require the use of a linearized filter, which has greater computational and implementation complexity. Instead, we utilize an error-state tracking Kalman filter, which only tracks the probability distributions of the errors building up for the different state elements [6].

The Kalman filter consists of two stages: predict and update. The predict stage is executed each time a new acceleration and angular velocity measurement are received, and the covariance of the different errors is updated according to the measurements. In parallel, the navigation equations [32] (integration and combination of the acceleration and angular velocity information to propagate the IMU state) are executed as normal, since we do not have correcting information yet.

We assume that the errors in gyroscope and accelerometer readings are Gaussian. The error variances are empirically determined to include the noise and scaling errors. The predict stage can be summarized by the error-state covariance update equation:

(1) $$P_{t|t-1}=F_{t}P_{t-1|t-1}F_{t}^{T}+\Sigma_{w}$$

where F is the error-state update matrix, P is the error-state covariance matrix, which tracks the probability distribution of the errors, and $\Sigma_{w}$ is the process noise covariance modeling the accelerometer and gyroscope errors. The standard Kalman filter equations also include the error-state ($\delta {\hat{x}}_{t}$) update equation; however, after each update stage, the error-state is applied to the actual IMU state and reset to zero; hence, tracking only the error-state covariance is sufficient.

The update stage is where the actual zero-v and zero-z updates (in short: zero-updates) are performed. The updated error-state $\delta {\hat{x}}_{t}$ and its updated covariance $P_{t|t}$ are calculated by:

(2) $$\begin{array}{cc} K_{t} & =P_{t|t-1}H^{T}{\left(HP_{t|t-1}H^{T}+\Sigma_{v}\right)}^{-1} \end{array}$$

(3) $$\begin{array}{cc} \delta {\hat{x}}_{t} & =K_{t}\left(0-H{\hat{x}}_{t}\right) \end{array}$$

(4) $$\begin{array}{cc} P_{t|t} & =P_{t|t-1}-K_{t}HP_{t|t-1} \end{array}$$

where H is the matrix that filters the state so that it contains only the elements that are corrected, 0 is a vector of all zeros (denoting the actual zero-updates), ${\hat{x}}_{t}$ is the predicted state calculated by the navigation equations before any zero updates and $\Sigma_{v}$ is the covariance matrix describing the small amount of uncertainty in the zero-updates. The error-state $\delta {\hat{x}}_{t}$, which denotes the difference between the predicted and corrected state of the IMU, is then subtracted from the predicted state ${\hat{x}}_{t}$ and immediately reset to zero.

### 5.3. Smoothing

The error-state tracking Kalman filter can produce accurate state estimations immediately after the zero-updates, while the estimation accuracy continues to degrade as the errors accumulate, until the next zero-updates. This is sufficient for a navigation application interested only in the absolute position; however, for the aims of this study, the evolution of the 3D position through each step (even when the foot is in motion) is also important.

To achieve this objective, we employ the Rauch–Tung–Striebel (RTS) smoother, which additionally performs a backward pass on the estimated state, and is initiated each time a zero-update is performed. The newly received correction information is propagated backwards to eliminate the accumulated errors, until the previous correction (previous zero-velocity phase) is reached. This operation introduces a total delay of a single step duration. Such a delay does not impair the near real-time property of the system, since a typical step is never longer than a few seconds. The smoother used in our system is based on [5].

**Figure 6:**
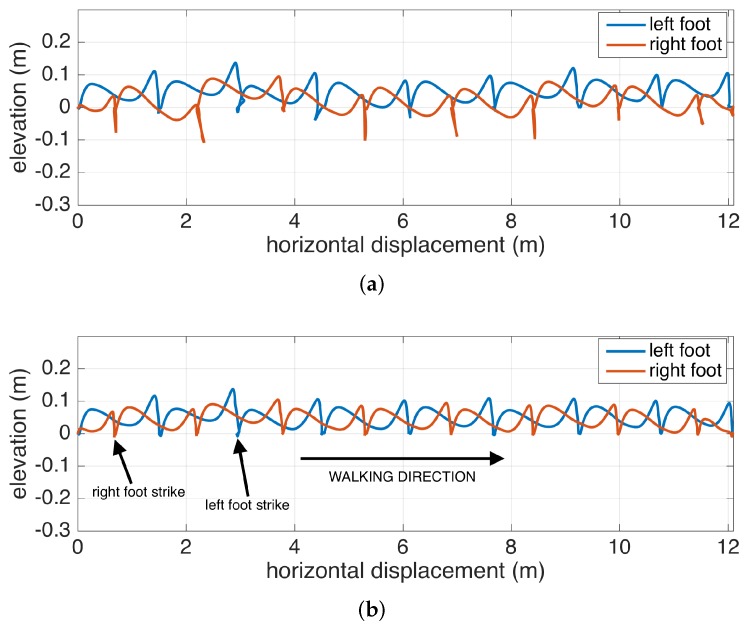
Lateral view of a walking subject: (**a**) only Kalman filtering, (**b**) Kalman filtering with Rauch–Tung–Striebel (RTS) smoothing.

The benefits of the RTS smoother are demonstrated in Figure 6, where it is compared with only Kalman filtering. The figure shows the feet position change of a subject walking to the right, when looking through the medial-lateral axis (i.e., looking from the side). The Kalman filter is able to accurately correct the position in each zero-velocity phase following the foot strikes, but the foot positions during the swing phases are subject to increasing position errors until a zero-velocity phase is reached (as observed in mid-swing positions that go below 0, which is floor-level). The backward passes executed after each zero-velocity period can accurately correct mid-swing errors.

### 5.4. Foot Orientation Estimation

A naive foot orientation estimation approach may be to directly compute it from the foot position change on the ground-tangential plane. This may yield accurate results if the subject is walking straight all of the time, but in reality, there are turning, side and even backward steps that would confuse this approach; though, it is not very unrealistic to assume that people walk straight and forwards most of the time and the forward steps usually cover more ground than side and back steps.

Based on this assumption, the problem of foot orientation estimation can be formulated as a problem of continuous parameter estimation, which should employ corrections with confidence proportional to the displacement (since steps with higher displacement are more likely to be forward steps). An option would be to use another Kalman filter for this purpose; however, Kalman filters require a linear state transition model, and in this case, it is non-linear since the relationship between displacement and heading is non-linear. An extended Kalman filter, which is the linearized version of the Kalman filter, would be a viable option; however, particle filters proved to be a better choice since as the error distribution of the foot orientation estimation grows, its shape deviates significantly from a Gaussian distribution; and particle filters are a good option to model such non-Gaussian error distributions. This is one of the reasons why particle filters are so popular in robot navigation literature, especially in odometry-based robot heading estimation studies, which bare many similarities to the problem at hand.

Before going into the formulation of the filter, it is important to remind that the error-state Kalman filter and the RTS smoother system is able to output accurate 3D position relative to the starting point, velocity and orientation change in the local frame. Hence, we can deduce the speed in the ground-tangential plane and the yaw change about the axis of the direction of gravity and use these information as input to the particle filter. What the Kalman filter does not know is the orientation of the foot relative to the computed positional change, which the particle filter is aimed to estimate.

The particle filter (PF) tracks the probability distribution of the underlying state by a cloud of numerous particles, subject to the same state transition and error models. Each particle has a state $x_{t}=\left(x_{t},y_{t},\theta_{t}\right)$ at time *t*, where $x_{t}$ and $y_{t}$ are the position of the particle on the horizontal plane (imagine looking top-down on a walking person’s foot), and θ is the direction of the particle. The particle propagation step updates the state of a particle by sampling the motion model distribution $P(x_{t}|x_{t-\delta t},v_{t},\delta \beta_{t})$, where $v_{t}$ and $\delta \beta_{t}$ are the speed and the yaw change, respectively, estimated by the error-state Kalman filter. The updated state of a particle is computed with the equations:

(5) $$\begin{array}{cc} \theta_{t} & =\theta_{t-1}+\delta \beta_{t}+\theta_{e} \end{array}$$

(6) $$\begin{array}{cc} x_{t} & =x_{t-1}+v_{t}\cdot \delta t\cdot \cos\left(\theta_{t}\right)+x_{e} \end{array}$$

(7) $$\begin{array}{cc} y_{t} & =y_{t-1}+v_{t}\cdot \delta t\cdot \sin\left(\theta_{t}\right)+y_{e} \end{array}$$

where $\theta_{e}$, $x_{e}$ and $y_{e}$ are small noises acting on different components of the state vector, drawn from a zero-centered Gaussian distribution. These noises are required to prevent particle depletion and premature convergence. The particle weights are then computed to be proportional to the output of a zero-mean Gaussian probability distribution function with the distance of the particle position to the position computed by the error-state Kalman filter used as input. The standard deviation of this Gaussian distribution is selected to be large (specifically, 2 m in this study), to allow the particle weights to remain considerably large until they are too far behind a significant displacement (to make use of the base assumptions). The particles are then resampled according to these weights.

At the end of each segmented step, the aggregate foot orientation is computed by taking the average of the particles’ directions and propagated backwards through the step via using the inverted $\delta \beta_{t}$ values, so that we can get a good continuous foot orientation estimate for the whole step duration.

**Figure 7:**
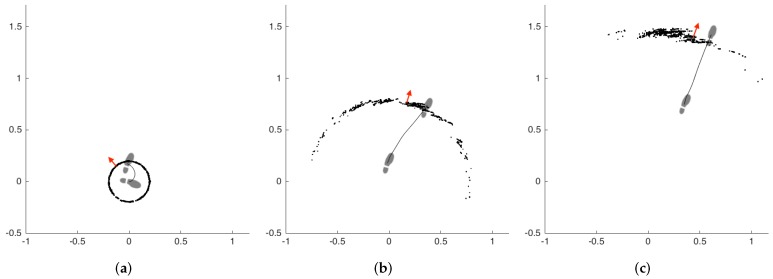
Example particle filter (PF) operation (footprints: true orientation; red arrows: estimation of PF). (**a**) first step, (**b**) second step, (**c**) third step.

A demonstrative example of the particle filter operation is presented in Figure 7. The subfigures represent three consecutive steps of the left foot, from left to right. The other foot is omitted for visual simplicity. The particles are denoted by black dots, and the aggregate foot orientation estimation of the filter is denoted by red arrows. The footprints show the true orientation of the foot. The first step is a side step with a turn of approximately 90 degrees. We selected this challenging case to demonstrate the capabilities of the filter better. The filter estimation after the first step is not accurate, but as the subject starts walking straight, the filter quickly converges on the true orientation.

It should be noted that this method aims to estimate the orientations of the two feet separately relative to the underlying displacement computed by the error-state tracking Kalman filter. Therefore, it computes the orientation changes relative to an arbitrary starting condition, as dictated by the underlying displacement data, and not the absolute orientation. The initial orientation state could be input to the system to allow this, if needed. Another alternative would be to utilize the magnetometer in the IMU to determine the absolute initial orientation of the sensor, although magnetometers are also subject to errors due to the magnetic interference in the environment. In this study, we employ neither of these approaches since the stride-wise relative orientation changes are sufficient for the purposes of this study, i.e., to be able to detect side and back steps in addition to forward steps, to estimate the correct rate of rotation along the estimated ankle rotation axis (which is input to the IC/FO algorithm) and to estimate the relative turning rate.

A drawback of this approach is that it neglects the toe out angle, which is defined as the natural outward alignment of the feet (i.e., the feet do not move exactly straight when lunging forward). This angle is typically about 20 degrees [7]. Since it stays almost unchanged throughout the gait cycle, this error’s effect on foot ankle rotation estimation proved to be insignificant; though, it is certainly in our future plans to incorporate other information (such as the principle component of the angular rotation, which is most likely to be around the ankle rotation axis) into the filter, which may enable us to estimate the toe out angle, as well [31].

### 5.5. Foot Ankle Rotation Axis and Rate Estimation

**Figure 8:**
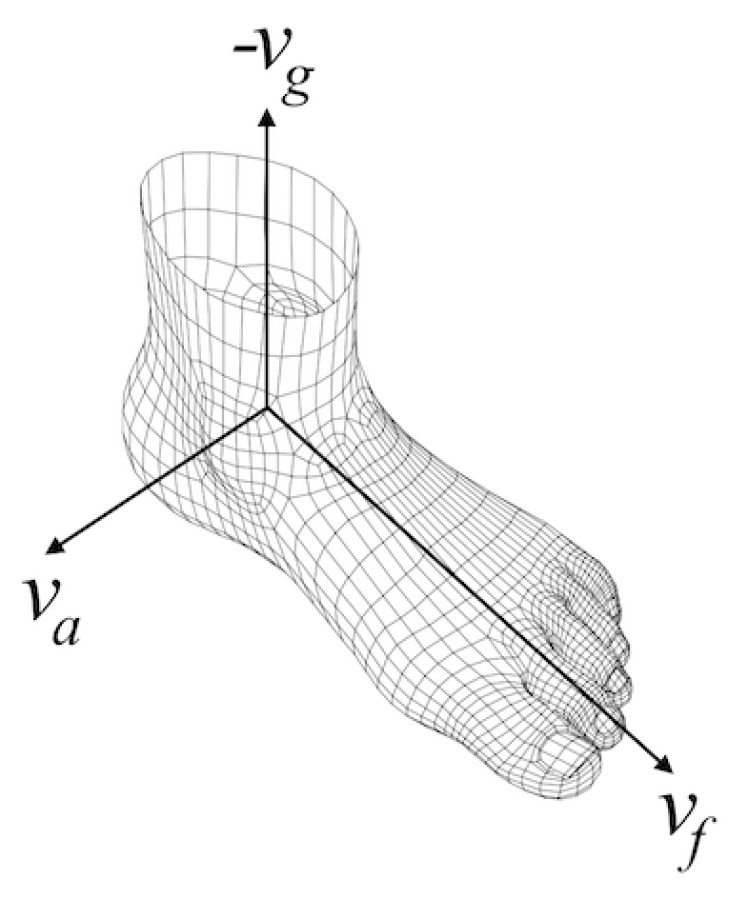
Foot-local coordinate system.

After we estimated the foot orientation vector ($v_{f}$) corresponding to the anterior-posterior axis, since we know the gravity direction vector ($v_{g}$), it is straight-forward to compute the ankle rotation axis ($v_{a}$) corresponding to the medial-lateral axis, by applying the cross-product to the former two vectors. What we get is a foot-local coordinate system with three perpendicular basis vectors, as shown in Figure 8.

Extracting the rotation rate about the ankle axis $v_{a}$ is a little trickier. The underlying error-state Kalman filter provides us with rotation matrices that map the sensor body coordinate system to the local coordinate system. These matrices can be converted to orientation changes of the sensor at each time interval in the local coordinate system (delta rotations). The problem is extracting the rotation around only the ankle axis and eliminating the remaining rotations around any other axes. Quaternions, which are one of the standard ways of representing 3D rotations, offer us a convenient way to do this. Therefore, we first convert the delta rotations to quaternions. Then, we use the following equations to project the delta rotation to the ankle axis:

(8) $$\begin{array}{cc} p & =\left(v\cdot v_{a}\right)v_{a} \end{array}$$

(9) $$\begin{array}{cc} q & =(p,w) \end{array}$$

(10) $$\begin{array}{cc} q_{a} & =q/∥q∥ \end{array}$$

where v and *w* are the vector and the scalar components of the input delta rotation quaternion, respectively, and $q_{a}$ is the resulting rotation around the ankle axis represented as a quaternion.

**Figure 9:**
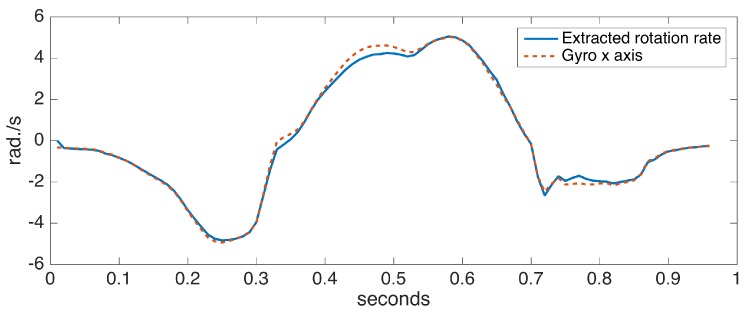
Extracted ankle axis rotation rate vs. gyro x axis of a correctly-positioned sensor.

To assess the accuracy of the proposed ankle axis rotation extraction method, we collected data from a sensor positioned on the foot with the x axis aligned with the ankle rotation axis (hence, fulfilling the assumption in [6]). We compared the x axis rotation rates with the extracted rotation rates. Figure 9 shows the comparison for a single step data, taken from a turning step (approximately 30 degrees) of the left foot. A turning step is deliberately chosen to make the case more challenging. The observed differences are minor, indicating the success of our approach.

### 5.6. IC/FO Events Detection

**Figure 10:**
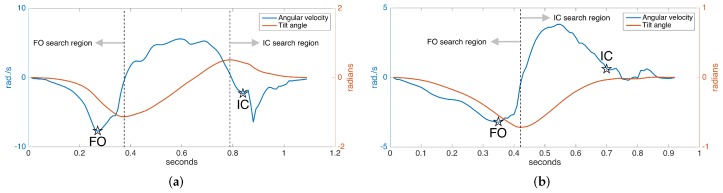
Detected FO and IC events: (**a**) normal gait and (**b**) pathological gait.

In normal gait, rotation around the ankle axis (in short, tilt angle) changes as follows, assuming we are looking from the lateral (right) side at a person walking towards the right: starting from the foot-flat phase, the foot begins to turn clockwise with the toe as the contact point; the foot leaves the ground (FO), and the clockwise rotation begins to slow down, then it reverses to counter-clockwise direction as the foot lunges forward; near the end of the forward motion, the toe elevation exceeds the heel elevation (toe lift), and the rotation reverses again to the clockwise direction followed by a heel strike. The tilt angles and the angular velocity (derivative of tilt angle) during the normal gait cycle are shown in Figure 10a. Notice there is one negative and one positive peak in the tilt angle, corresponding to the times that the angular change reverses direction. These instances correspond to right after the FO and right before the IC, respectively. Hence, they can be used to limit the FO and IC search regions, since it is guaranteed that there can be no FO or IC events in-between [7].

In [6,31], it is presented that the two prominent negative peaks in the angular velocity mark the FO and IC events with sufficient accuracy. This means that the foot reaches maximum angular speed as it leaves the ground. Our approach is also based on this observation. However, around the IC event time, we usually observe two consecutive negative peaks, the first one due to the impact of the heel strike and the second one due to the angular speed reaching its maximum right after the heel strike. Therefore, if these two peaks can be detected, the first one marks the IC [6]. An example of the detected FO and IC events is presented in Figure 10a.

While the observations above are valid for normal gait, they are partially true for pathological gait. Especially for Parkinson’s patients in later stages, the heel strike is not observed, as the foot strikes the ground all at the same time (in some extreme cases, even the toe may strike first). Hence, there is no toe lift to enforce the heel to be the first contact point. This means that there is no positive peak in the tilt angle, and the negative IC peaks in the angular speed may not be observed (Figure 10b). Therefore, the previously outlined algorithm is not valid for detecting the IC event in such cases and has to be extended.

Since in the absence of toe lift, the foot strikes the ground almost all at once, we may use the foot elevation information to detect if the foot has contacted the ground. Thankfully, the underlying error-state Kalman filter provides us with this information. Therefore, if we detect no toe lift, we may find the IC event by detecting the instance when the foot elevation drops to a substantially low level (e.g., a few millimeters). An example of the FO and IC events detected in the case of pathological gait is presented in Figure 10b. Note that the swing times (duration between FO and IC) in this case are much shorter than the normal case, as expected.

Once we have detected the FO and IC events, it is straightforward to determine the two major gait phases: swing time and stance time. Swing time corresponds to the period starting from the FO and ending at the IC, and the stance time vice versa.

### 5.7. Detection of Turning Steps

Extracting the foot orientation has another benefit: the ability to detect turning steps and extract other metrics specific to them. We employ a simple method for detecting turns: we try to find consecutive turning steps (with angle changes from start to end of a step exceeding a certain threshold). Since our test scenarios include 180 degree turns at the ends of a corridor, we specifically aim to detect these turns, which are especially challenging for patients experiencing pathological gait.

**Figure 11:**
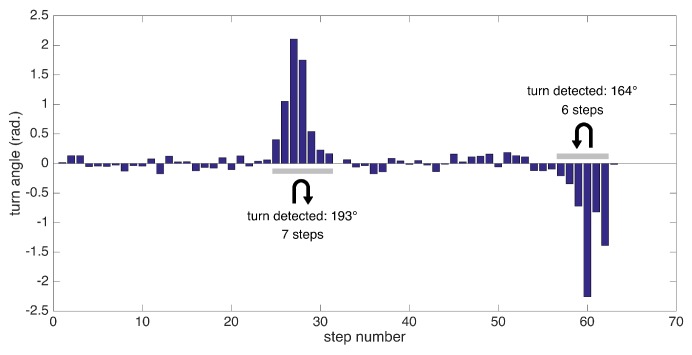
Turn angles for individual steps and detected substantial turns.

In summary, we can detect the following metrics related to turning: the number of steps to complete a specific turn (depending on the data collection session) and turning rate (degrees/s). An example of substantial turns’ detection is presented in Figure 11.

### 5.8. Extraction of Spatio-Temporal Gait Metrics

After the IC and FO events are detected, their time instances can be used to determine stance and swing times. The IC event times can further be used to segment strides, making the connection between the spatial and temporal elements of gait, which in turn enables the extraction of stride length, clearance, cycle time, cadence and speed. The metrics of stance and swing times, stride length and clearance are computed per stride and per foot, and hence, their variabilities (standard deviation) are also computed. Turning rate is a direct output of the turning steps’ detection module, as the total turn angles are divided by the total amount of time it takes to complete a turn.

### 5.9. Average Lateral Stride Profile Visualization

Apart from the numeric gait metrics, a visual representation of the average lateral stride profile (looking through the medio-lateral axis) for each foot is also beneficial to assess the position evolution of the feet through a step. The aim is to process all of the strides taken during a data collection session and aggregate them into an easily-comprehendible visual representation for both feet. The lateral view provides us with a glance at both the stride length and the clearance (elevation) changes during the swing phase. Being able to see average profiles separately for each feet also allows the clinicians to spot any clearance asymmetries.

Changes in the walking speed and stride length in a session do not necessarily point to gait abnormalities, as people tend to modify these at will when walking in a leisurely mode. Hence, the durations of individual strides may vary. Therefore, simply taking the average of position values belonging to the same time instance does not yield the intended results, as it tends to smooth out the secondary peaks (corresponding to the toe-lift just before IC) in the case of strides with different lengths. This information loss can be averted by taking a solely spatial approach rather than incorporating time information: we select a specific number of points (typically high) on the individual stride profiles that are distributed uniformly, as in the neighboring points, which are equidistant. Then, we average the points that correspond to the same index and achieve spatial aggregation. This way, both the primary (elevation right after FO) and the secondary (toe lift just before IC) peaks are preserved. To show the elevation variability among individual steps, we also visualize the standard deviation curves as dashed lines. Consequently, we incorporate as much spatial information as possible into an uncluttered and easily readable figure.

**Figure 12:**
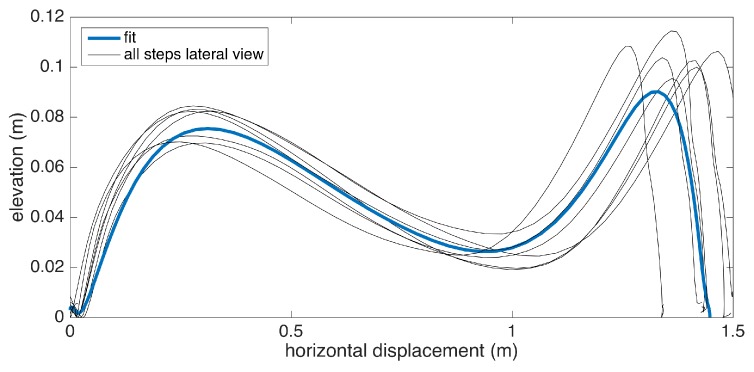
Example average stride profile fit over individual lateral stride profiles.

An example is presented in Figure 12. The individual stride profiles are also drawn to give an idea on the underlying data that are used to generate the average profile.

**Figure 13:**
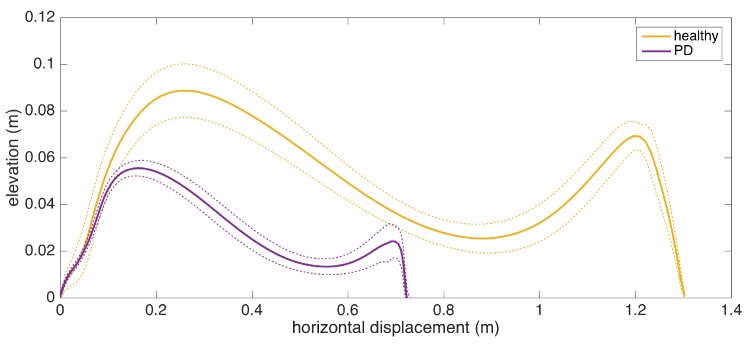
Comparison of average stride profiles of healthy and PD subjects.

To give an insight into how the visualization may be used for clinical assessment purposes, we also provide a real example that compares a healthy elderly person with a PD patient (Figure 13). Even though comparing abnormal and normal gait is not within the aims of this study, such an extreme case highlights the type of differences that the visualization may reveal. Both subjects are male, and their ages are similar (76 and 83, respectively). The first difference to note is the big difference between stride lengths. Another important difference is that the secondary peak (right-most) in the PD patient is much lower compared to the first peak, indicating a difficulty performing the toe lift at the end of the swing phase. This is typical of PD patients and in severe cases, the toe-lift completely disappears, meaning that the subject places the foot all at the same time without a pronounced heel strike. Both of these symptoms describe what is referred to as the Parkinsonian shuffling gait, which is commonly observed in PD patients at later stages.

## 6. Experiments

We conducted a two-phase experimental study to validate the numerical accuracy of the extracted metrics and to demonstrate what the metrics can tell us clinically. In the initial system validation phase, we compare the stride length and swing time estimation performance of our system with a reference system (IR depth-camera-based gait analysis system supported by a slow motion camera). The data used for validation is comprised of 22 subjects including healthy subjects and patients of various neurological disorders. The results are presented in the next section. In Section 6.2, we present the extensive output of our system from a disjoint dataset of 17 patients, and we discuss the clinical significance of the extracted metrics with the interpretations of a neurologist. With these two experimental studies, we aim to show the usability of our system, both technically and clinically.

All of the subjects included in this section gave their informed consent for inclusion before they participated in the study. The study was conducted in accordance with the Declaration of Helsinki, and the protocol was approved by the Ethics Committee of Bogazici University Institutional Review Board for Research with Human Subjects (Identification Code 2015/56).

### 6.1. Comparative System Evaluation with Kinect and Slow Motion Camera

To assess the numerical accuracy of the extracted metrics, we conducted a preliminary evaluation study to compare the output of our system with a system based on a Microsoft Kinect depth-camera (for stride length comparison) and a slow motion camera (for stance and swing times comparison). The accuracy of the Microsoft Kinect depth-camera [34] for gait analysis purposes has been well investigated in the literature [35,36,37,38]. Especially for estimating stride length, good validation results have been reported when compared with the gold standard instrumented walkways and camera-based motion capture systems: in [35] and [38], mean absolute displacement errors of 2.5 cm and 2.9 cm per stride are reported, respectively. The applicability of Kinect as a tool for analyzing pathological gait, particularly PD, is also explored in [39,40,41], with good results. Considering these characteristics, the Microsoft Kinect serves as a viable alternative that can be deployed in a non-hospital setting for estimating stride lengths. However, it should be noted that the output of the Kinect system still cannot be treated as the ground-truth, as opposed to a gold standard system. Therefore, in our validity analysis, we employ an agreement analysis methodology, namely Bland–Altman and correlation plots, that does not treat the Kinect as the absolute reference.

Even though the Microsoft Kinect is a good gait analysis system alternative, it does not provide the same level of portability and convenience as an IMU system does: it still requires infrastructural setup (a tripod to mount the device, a power source and a wired connection to a computer for data collection), and its operational range is limited to 1.5–4 m, where the IR depth sensors are the most accurate [37]. Hence, we employ the Microsoft Kinect only for validating the spatial accuracy of the IMU-based system and not as a part of the core system.

We specifically used the Microsoft Kinect v2 camera, mounted on a tripod at a height of 0.7 m, to record the patients moving through a corridor towards the camera (along the anterior-posterior). We only considered strides within the most accurate operational range of 1.5–4 m, since we want the Kinect data to be as reliable as possible to be used as a reference. The spatial accuracy of the Kinect data further increases during the periods where the feet are stationary (i.e., the zero-velocity periods). Hence, extracting the displacement (computed via the depth readings) between two consecutive zero-velocity periods of a specific foot yields very accurate stride length estimations.

For validating the temporal metrics, such as swing, stance and cycle times, the Kinect system proved to be not accurate enough. For instance, in [35], absolute cycle time errors of 0.24 s are observed. Therefore, we decided to additionally employ a slow-motion camera (recording at 240 frames per second) placed on the floor beneath the Kinect camera. We conducted a frame-by-frame analysis of the recordings to determine the IC/FO times and consequently determined swing, stance and cycle times for each stride. It should be noted that the accuracy of the detected IC/FO times directly determines the accuracy of the computed swing and stance times, since they are defined as the complementing periods between consecutive IC and FO events (see Section 2 for detailed definitions).

We collected validation data from a total of 22 subjects, 16 of whom (8 males, 8 females) are healthy, and the remaining 6 (3 males, 3 females) suffer from different neurological conditions with gait implications; particularly, two subjects with frontotemporal dementia, one with vascular dementia, one with vascular mild cognitive impairment, one with normal pressure hydrocephalus and one with Parkinson’s disease. The healthy subjects’ average age is 43.9±15.7 (range: 25–76), and the average height is 1.68±0.08 m. The subjects with neurological conditions have an average age of 50.6±18.3 (range: 52–79) and an average height of 1.67±0.08 m.

Whenever possible, the subjects were instructed to strap the IMUs on their feet themselves, with minimal intervention, to increase the sensor placement variability. The subjects were then asked to walk in the designated test area at their preferred pace. Three to 5 sessions were conducted for each subject to increase the amount of data.

To demonstrate the correlation and the agreement between the reference system and our system, we generated correlation and Bland–Altman plots [42], which is a standard method of analyzing the agreement and the differences between two measurement methods [43]. The method differences are plotted against the averages, together with the limits of agreement (LOA), corresponding to the 95% confidence interval of the differences. We first utilized the Kolmogorov–Smirnov test to ensure the normality of the data before the analyses.

**Figure 14:**
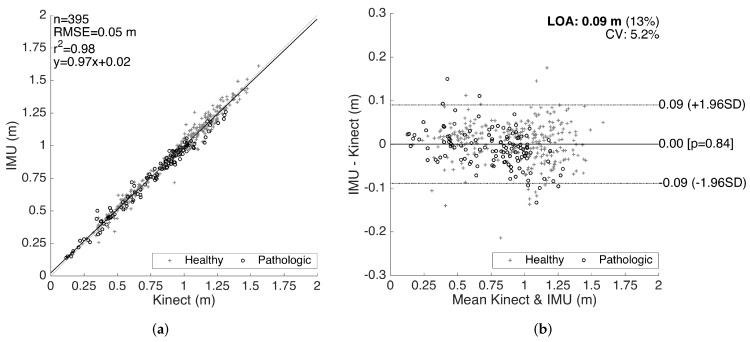
Stride length validation. (**a**) correlation plot and (**b**) Bland–Altman plot.

The results for the validation of stride lengths is presented in Figure 14. The healthy and pathological data are plotted with separate markers. The linear regression on the correlation plot demonstrates a good fit with $r^{2}$ = 0.98, and the slope of the fit (0.97) shows that there is no significant bias proportional to the magnitude of the measured quantity. The root-mean-square error (RMSE) is 0.05 m. The LOA are on average 0.09 m per stride, which is clinically acceptable. Moreover, there is no significant bias (mean of differences is $4.6\times 10^{-4}$ m). The characteristics of the method differences also do not change significantly over the range of the measurements, indicating no considerable proportional bias. There is also no significant difference between the error characteristics of the healthy and pathological data.

**Figure 15:**
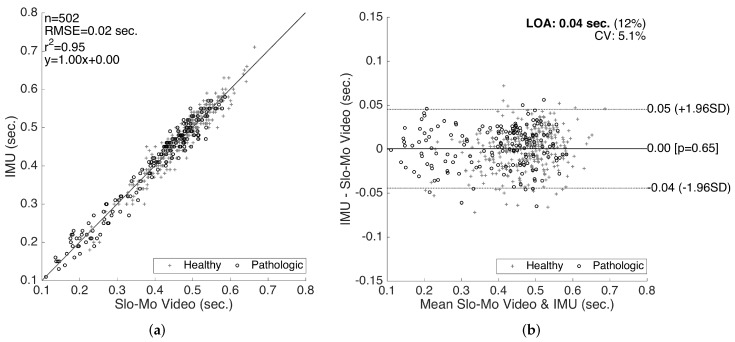
Swing time validation. (**a**) correlation plot and (**b**) Bland–Altman plot.

The results for the validation of swing times are presented in Figure 15. Even though we also compiled the validation results for the stance times (and consequently the cycle times as the sum of the two), in this paper, we only present the validation results for the swing times, since the stance time errors are determined to be proportional to the swing time errors (in the opposite direction), as they complement each other to form a cycle. Similar to the stride length validation results, a good correlation fit (with RMSE = 0.02 s, $r^{2}=0.95$ and slope of 1.0) is observed. There is no considerable bias (mean of differences is $4.7\times 10^{-4}$ s) or significant changes in the error characteristics over the range of the measurements. The LOA (on the average 0.04 s) are clinically acceptable. Similar to the stride length validation results, there is no significant difference between the accuracy of the healthy and pathological data, indicating that our methods work reasonably well for abnormal gait, as well.

### 6.2. What Can the Metrics Tell Us?

To assess the clinical significance of the extracted metrics, we collected a separate dataset from 17 patients of various neurological conditions during outpatient visits and compared the results to the actual clinical findings. We also assembled a control group from the 16 healthy participants of the validation study. We first present the discussion on the control group results, which will serve as a reference for the gait abnormalities observed in the neurological disorders group.

#### 6.2.1. Healthy Control Subjects

The control group is composed of 16 healthy subjects (8 males, 8 females), who also participated in the validation study presented in Section 6.1. The tests consist of multiple (3–5) sessions of walking approximately 5 m along a straight path, for each subject.

The extracted gait metrics of the control group are presented in detail in Table 3. The gender, age and height of each subject are also presented. This data may be treated as a reference for assessing the gait abnormalities of the neurological disorder subjects discussed in Section 6.2.2. The summary of the significant control group findings is as follows:

- Stride length is affected by age, height and gender, as well as the intended walking speed of the subject at the moment. Therefore, it is not possible to determine gait abnormality solely by looking at this metric; it has to be assessed with respect to the subject features and other metrics. However, extreme low values may indicate abnormality. The average stride length among the control subjects is 1.16±0.14 m, with the lowest observed value being 0.93 m. The average is well above 1 m, and extreme low values are uncommon. Therefore, the interpreter should look out for extreme values rather than small inconsistencies.
- Cadence is similar to stride length, as it is also affected by by age, height, gender and the subject’s intended pace. However, the effects are much weaker, suggested by the low variability (the average is 93.6±6.34). As a person attempts to increase his/her pace, both the average stride length and cadence increase. Therefore, these two metrics should be interpreted with respect to each other. For instance, a low stride length value combined with high cadence might indicate the attempt to achieve a certain speed by compensating the small steps by a faster rate, which may be a sign of abnormality. The combined effect of these two metrics is reflected in speed. Cycle time is inversely proportional to cadence; thus, similar observations can be made.
- For the metrics that have separate components for each side (left and right), the most important observation for the control subjects is that there are no considerable asymmetries. The normal gait is left-right symmetric, and this property is reflected in the metrics. Asymmetries are mostly observed in the stance ratios and max. clearance values, and these two metrics usually also affect each other.
- In addition to the similarity of the left and right stance ratios (no asymmetry) for the control subjects, we also observe that they are very close to the normal stance ratio value of 0.6 over 1 (60%). The averages are 0.61±0.021 for the left side and 0.61±0.026 for the right side, with the variability being very low. Any significant deviation from the nominal value may indicate abnormality.

#### 6.2.2. Neurological Disorder Subjects

The dataset consists of data collected from 17 subjects (average age: 78±10.7) in a private clinic setting. Eight of these subjects suffer from Parkinson’s disease (PD); two have dementia with Lewy bodies (DLB); one has mild cognitive impairment (MCI); one has vascular dementia (VaD); one has normal pressure hydrocephalus (NPH); one with vascular Parkinsonism (VaP); and the remaining three suffer from multiple conditions. The individual condition, age, gender and height of the subjects are presented in Table 4. Furthermore, we assigned a subject ID to each for easy reference.

The data collection is supervised by the neurologist and is designed to not interfere with the regular neurological examination procedure. The tests consist of one or more sessions of walking along a corridor of 8 m in length and performing 180 degree turns at the ends of the corridor, for each subject. A session is terminated when the subject gets tired or a sufficient number of laps (typically three) has been completed. This procedure is not different from the regular gait assessment procedure of the neurologist; hence, no extra effort/time was required of the neurologist other than strapping the IMUs before the walking sessions and initiating the data collection.

The list of the extracted metrics for all of the neurological disorder subjects is compiled in Table 4. The metrics that point to a possible gait abnormality are highlighted as bold. The standard deviation values for the metrics that are averaged over multiple strides are also available, but omitted due to space constraints. In the discussions of the individual subjects below, the standard deviation values will be provided if they indicate a clinical finding. Usually, we aggregate multiple sessions of a subject into one if the sessions are conducted back-to-back and the gait characteristics do not deviate from session-to-session. However, for the patient with ID 17, we decided to compile the metrics separately, as two different sessions correspond to PD medication ON and OFF states, respectively. The results of this subject are discussed in more detail in the upcoming text.

When interpreting the metrics, it is important to consider multiple related metrics together, as focusing on a single metric might be misleading. Some metrics for the neurological disorder subjects overlap with the metrics of the control group when viewed individually, but abnormalities may be detected when a metric is assessed together with other metrics. For instance, a low stride length value may not necessarily indicate an abnormality, as the subject may be intentionally walking slowly. However, if we observe a high cadence value combined with a low stride length, it could indicate that there is a difficulty lifting the feet off the floor, and the subject is trying to compensate by taking steps faster. Another example would be the stance and swing times. Looking at these metrics individually would not tell us much; instead, their ratio should be assessed (as reflected in the stance ratio metric). The metrics that have right and left counterparts, such as maximum clearance, should also be considered with respect to each other. An overall low clearance would not indicate an abnormality, since it is also affected by the pace or the intentional sure-footedness of the subject. An asymmetry between left and right may indicate a gait problem though.

Subject 1 has DLB and vascular mild cognitive impairment. There were no clinical findings of gait abnormality, and the metrics reflect that.

Subject 2 has DLB. There is a slight temporal asymmetry with right and left feet stance ratios being 60 and 65%, respectively. The cadence is higher compared to a leisurely walk, but the stride length is within the normal range, suggesting that the subject walked fast intentionally. None of these findings point to a considerable gait problem, which is also verified by the clinical observation.

**Figure 16:**
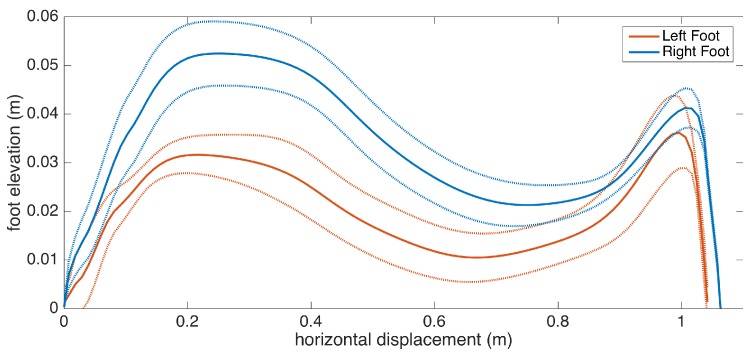
Feet elevation (clearance) asymmetry (Subject 3).

Subject 3 has PD pronounced on one side, as the clinical observation for this patient is the left-right asymmetry. This observation is reflected in the metrics, as there is significant feet clearance asymmetry (left: 3.9 cm, right: 5.3 cm), shown in Figure 16, and a difference between the left-right stance ratios.

Subject 4 has PD with classical Parkinsonian gait. The overall muscle rigidity causes magnetic feet, as reflected in the high stance ratios (72%) compared to the typical healthy stance ratio of 60% [7]. The stride lengths and feet clearance are also low (50 cm and 4.5 cm respectively), which are also consistent with Parkinsonian gait.

Subject 5 has VaD and DLB, without any extra-pyramidal symptoms, but with overall slow movement. There is no significant asymmetry as also confirmed by the clinical observation. The slowness is reflected in the cadence, speed and turning rate metrics.

Subject 6 also exhibits Parkinsonian gait. Low stride lengths, high stance ratios and low turning rate are observed. There is also left-right asymmetry as seen in the uneven feet clearance values, as confirmed by the neurologist.

Subject 7 has been diagnosed with NPH, showing problems in maintaining balance. This is reflected in the low turning speed while the other metrics do not show any considerable abnormalities.

**Figure 17:**
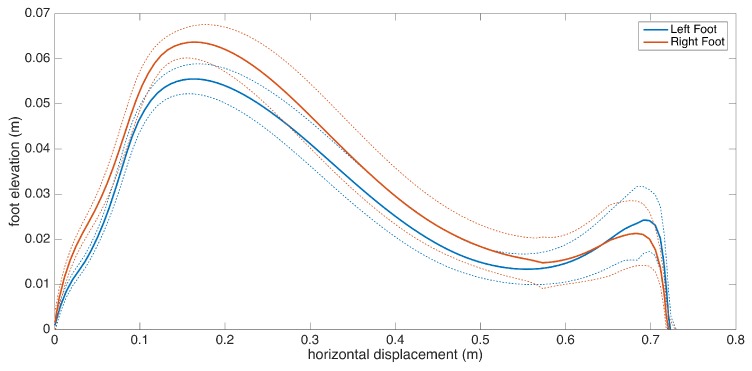
Even footfall as demonstrated by lower secondary peaks (Subject 8).

Subject 8 also exhibit Parkinsonian gait. The movement is slow overall, as shown by low stride length, cadence and speed values, as a result of muscle rigidity. This patient also has even footfall with less pronounced heel strikes, which is also consistent with Parkinsonian gait, as demonstrated in the lateral stride profile (Figure 17).

Subject 9 suffers from VaD, DLB and also shows Parkinsonism. The stride lengths are low, contributing to the overall slow movement. The stance ratios are slightly higher than normal, with right stance times slightly higher than left, combined with decreased feet elevation.

Subject 10 has VaD with no gait-related symptoms. The metrics also do not indicate any abnormality.

Subject 11 is a PD patient in the medication OFF state, showing typical gait symptoms of PD, such as decreased stride lengths, even footfall and right-left asymmetry (both indicated by the feet clearance and stance ratios). One important observation is the high cadence values, which indicate that the patient tries to compensate decreased stride lengths with frequent steps, as also clinically observed.

Subject 12 is also a PD patient exhibiting gait-related symptoms similar to Subject 11. However, stride lengths are even smaller, as also affected by the subject’s age. There is a slight stance ratio asymmetry (left: 66%, right: 62%), confirming the clinical observation of slightly higher left-sided rigidity.

Subject 13 has PD with no considerable gait-related symptoms, as also supported by the gait metrics, which show no considerable abnormalities.

Subject 14 also exhibits no gait-related symptoms as expected from MCI. The metrics are consistent with this.

Subject 15 suffers from VaP with Parkinsonism symptoms. The magnetic feet are indicated by the high stance ratios and low feet clearance values. The stride lengths are very low. Left heel strikes are absent (even footfall), which is consistent with the clinical observation of slight left dominance of the extra-pyramidal symptoms.

Subject 16 has DLB, causing left-right gait asymmetry, as indicated in the higher left stance ratio. No other gait abnormalities are observed.

We were able to collect medication ON and OFF state data from Subject 17, who is a PD patient. The ON and OFF sessions are denoted by Subject IDs 17-ON and 17-OFF in Table 1, respectively. The data collection times were two hours apart. The medication ON state denotes the period during which the medication is active and the symptoms are mitigated. Assessing how and to what extent the symptoms are mitigated is especially important to be able to adjust the medication dosage of PD patients quickly and accurately. Furthermore, excess dosage may cause motor complications, such as dyskinesia, which further increases the significance of monitoring ON/OFF periods. The typical procedure for clinicians to assess medication effects is the feedback of the patient or the caregiver, which may be subjective and hence require a few weeks to collect and validate. Our system provides a faster and more objective method.

**Figure 18:**
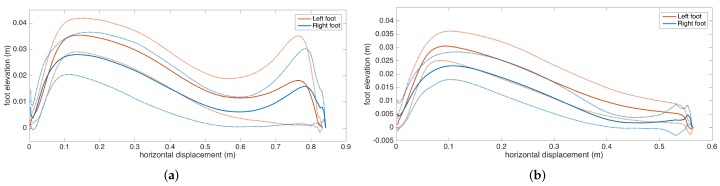
Lateral step profile of a PD patient (Subject 17). (**a**) ON state and (**b**) OFF state.

**Figure 19:**
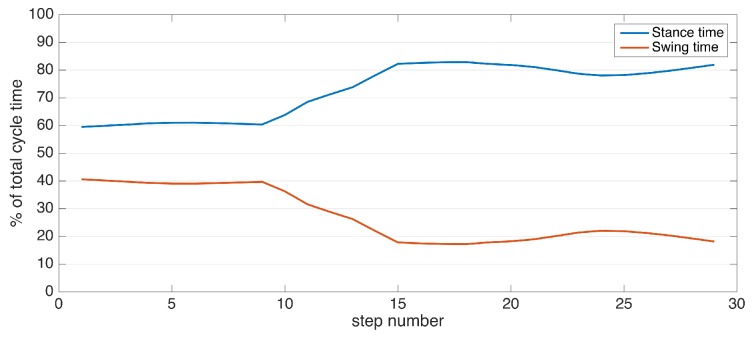
Change of stance and swing time ratios due to fatigue (Subject 17).

The ON state lateral step profile is presented in Figure 18a. Even though the feet elevation and stride lengths are generally low, there is still a pronounced heel strike as the patient can still lift her toes before the IC. However, as fatigue kicks in about the middle of the session, gait becomes more dysfunctional. The toe lift decreases (as reflected in the increased standard deviation curves towards the secondary peak of the step profiles). This behavior is also apparent in the change of the stance and swing time ratios through the session, presented in Figure 19. The stance times get longer as the feet of the patient become harder to lift off the ground. In the OFF state, the heel strikes disappear completely (Figure 18b), and the feet are stuck on the ground for extended durations (indicated by the high stance ratios and low feet clearance values), consequently leading to foot dragging.

## 7. Conclusions

In this study, we proposed a robust IMU-based gait analysis system suitable for non-hospital settings that is able to extract a rich set of standard spatio-temporal gait metrics. The system is completely mobile with no requirement of an infrastructural element. The extracted metrics are supported by a novel lateral stride profile visualization method, which enables the instant visual interpretation of the underlying spatial gait characteristics.

We conducted a two-phase experimental study to validate the accuracy of our system and to provide individual case reports of various neurological disorders that demonstrate the system’s clinical applicability. The results indicate that our system can indeed capture various neurological disorder-related gait abnormalities in a non-hospital setting.

As future work, we plan to extend the capabilities of our system by devising and extracting higher level metrics (e.g., metrics that quantify asymmetries). Furthermore, in the future, we wish to increase our patient base and to conduct an extended study with more clinical content, including the systematic analysis of the gait-related differences/correlations between various subject groups, such as young healthy, healthy elderly, Parkinsonism and other neurological and possibly orthopedic conditions. We are also aware that our system validation study is preliminary, as it lacks comparisons with a gold standard reference system. Therefore, to strengthen the clinical validity of our system, we plan to conduct more experiments that compare the numeric gait metric results to a reference system installed in a non-hospital setting.

## Figures and Tables

**Table 1:** Spatio-temporal gait metrics.

Gait Metric	Definition
Stride length (m)	Distance between two successive placements of the same foot.
Step length (m)	Distance by which a foot moves in front of the other foot. The sum of two successive step lengths corresponds to stride length.
Walking base (cm)	Side to side distance between the motion lines of the two feet.
Cadence	Number of steps taken per minute.
Cycle time (s)	Duration of a single stride. Cycle time is inversely proportional to cadence and hence can be used as an alternative to it.
Stance time (s)	Duration of stance phase, starting with initial-contact (IC) and ending with foot-off (FO) of the same foot.
Swing time (s)	Duration of swing phase, starting with FO and ending with IC of the same foot.
Speed (m/s)	Stride length/cycle time.
Clearance (cm)	elevation of the foot during the swing phase. This metric can be diversified as minimum and maximum foot clearance (elevation).
Turning rate (degrees/s)	Rate of the direction change of a foot. Positive values are normally observed during turning steps.

**Table 2:** Related work on gait analysis with IMUs.

Ref.	Aim (*Methodology*)	Sensors	Subjects	Strengths (Novelty)	Weaknesses
[12]	Body joint angles’ estimation (*3D-orientation differences between sensors*).	Force sensors integrated in shoes; IMUs: 2 on ankles, 2 on knees, 1 on trunk	1 healthy	Multiple sensors of different modalities enabling accurate joint angles information.	Only demonstrative results for a single subject (proof of concept). No gait metrics extracted. Relatively higher cost.
[6]	IC/FO detection (*peak detection*), stride length estimation (*Kalman filtering with zero-velocity updates*).	2 IMUs on feet	16 PD patients (ON state)	High spatial accuracy due to Kalman filtering methodology. Real patients.	IC/FO detection does not consider pathological gait. The patients are in the ON state. No control group.
[13]	Calibration to mitigate sensor misplacement (*procedure with a set of specific movements*), stride length estimation (*double integration*).	2 IMUs on ankles	8 healthy	Considers sensor placement errors and proposes a procedure to mitigate them.	Low spatial accuracy due to double integration. Only healthy subjects.
[14]	Drift removal in gyroscope signals. Estimation of cycle time, stride length, step length and cadence (*geometric human locomotion model*).	IMUs: 1 on pelvis, 2 on thighs, 2 on shanks, 2 on feet	5 healthy	A comprehensive system with numerous sensors to extract a rich set of gait metrics. Drift removal to increase accuracy.	Only healthy subjects, not targeted towards gait abnormalities. Obtrusive due to the number of sensors.
[15]	IC/FO detection (*peak detection*).	IMUs: 2 on shanks, 2 on feet	13 spinal cord injury patients, 12 healthy	Considers pathological gait. Experiments with real patients.	Obtrusive: the sensors are wired to a PDA; the sensors on shank are attached to the skin via double-sided tape. No spatial metrics.
[16]	IC/FO detection (*peak detection*), stride length estimation (*geometric human locomotion model*).	IMUs: 2 on forearms, 2 on shanks, 2 on thighs	10 PD patients, 10 healthy	Rich set of spatio-temporal metrics. Real patients.	Low accuracy in spatial metrics. Obtrusive due to number of sensors.
[17]	Analyzing changes in spatio-temporal metrics prior to freezing of gait. IC/FO detection (*peak detection*), stride length estimation (*double integration*).	2 IMUs on ankles	5 PD patients	Rich set of spatio-temporal metrics. Real patients.	Low accuracy in spatial metrics. Limited number of subjects. No control group.
[18]	IC/FO detection (*peak detection complemented with HMM*).	1 IMU alternated between various placements	1 healthy	A solution adaptive to a variety of placements.	Not targeted for pathological gait. Only one healthy subject.
[19]	Estimation of cycle time and stride length (*geometric human locomotion model*).	IMUs: 2 on shanks, 2 on thighs	6 PD patients, 7 healthy	Real patients.	Low accuracy in spatial metrics. Stance/swing times are not extracted (only cycle time).
[20]	Estimation of stride length and stride velocity (*geometric human locomotion model*).	2 IMUs on shanks	10 PD, 36 hip-replacement, 7 coxarthrosis patients, 18 healthy	High number of real patients with multiple different conditions affecting gait.	The datasets belong to previous studies and are not collected by the authors. Limited temporal metrics (only stride velocity).
[21]	IC/FO detection (*force sensors*), stride length estimation (*double integration*).	Force sensors and an IMU integrated in a shoe	5 PD patients, 10 healthy	Different modalities integrated in a shoe. Real patients.	Low spatial accuracy due to double integration. Relatively higher cost.
[22]	IC/FO detection (*peak detection*), stride length estimation (*double integration aided with zero-velocity updates*).	2 IMUs on feet	10 healthy	Increased spatial accuracy due to zero-velocity updates (compared to only double integration).	Obtrusive system: The sensors are wired to a control node. Only healthy subjects.
[23]	Classification of different movement patterns, estimation of stance/swing times (*naive Bayes classifier*).	Force sensors and an IMU integrated in an insole	5 healthy	Ability to detect different types of steps including lateral and backward walking.	Only healthy subjects, not targeted towards gait abnormalities. Relatively higher cost.
[24]	Detection of heel strikes (*peak detection*), asymmetry indices from raw acceleration intensity (*spectral analysis*).	1 IMU on trunk	15 healthy	Asymmetry indicators computed from raw data.	Only healthy subjects. No standard spatio-temporal metrics extracted.
[25]	IC/FO detection (*peak detection*), balance detection (*raw acceleration signal processing*).	IMUs: 2 on feet, 1 on waist	21 AD patients, 50 healthy	Balance features that indicate the intensity of lateral sway.	IC/FO detection methodology is not novel. No spatial metrics extracted.
[26]	Regularity and symmetry indices (*Fourier analysis on raw acceleration signals*).	1 IMU on waist	64 PD patients, 32 healthy	The high number of real patients.	The methodology is not clearly explained.
[27]	IC/FO detection (*wavelet-based time and frequency analysis*), stride length estimation (*inverted pendulum model with double integration*).	1 IMU on trunk	30 PD patients, 30 healthy	High number of real patients.	Low accuracy in spatial metrics.
[5]	3D feet position estimation (*Kalman filtering with zero-velocity updates, RTS smoothing*).	2 IMUs on feet, 1 camera on foot for spatial sync.	1 healthy	High spatial accuracy.	No standard spatio-temporal metrics extracted. Not targeted for pathological gait.

**Table 3:** Control subjects and extracted gait metrics.

Subject ID	Gender	Age	Height (m)	Stride Length (m)	Cadence (steps/min)	Cycle Time (s)	L Stance Time (s)	R Stance Time (s)	L Swing Time (s)	R Swing Time (s)	L Stance Ratio	R Stance Ratio	Speed (m/s)	L Max. Clearance (cm)	R Max. Clearance (cm)
C-1	F	28	1.70	1.13	92.9	1.29	0.84	0.74	0.50	0.51	0.63	0.59	0.87	7.3	7.2
C-2	F	49	1.55	1.17	103.6	1.16	0.74	0.70	0.44	0.44	0.63	0.61	1.01	8.5	8.3
C-3	M	26	1.72	1.11	96.8	1.24	0.73	0.76	0.51	0.49	0.59	0.61	0.89	8.2	8.1
C-4	M	27	1.82	1.44	96.2	1.25	0.77	0.70	0.53	0.50	0.59	0.58	1.15	8.2	8.8
C-5	F	25	1.68	1.16	92.1	1.30	0.82	0.80	0.47	0.52	0.64	0.61	0.89	8.5	8.5
C-6	M	34	1.76	1.14	95.1	1.26	0.77	0.80	0.47	0.50	0.62	0.62	0.91	8.2	8.1
C-7	M	29	1.73	1.22	92.1	1.30	0.82	0.84	0.50	0.45	0.62	0.65	0.94	9.3	8.9
C-8	M	39	1.65	1.18	105.0	1.14	0.70	0.71	0.44	0.44	0.62	0.62	1.03	8.7	8.6
C-9	M	36	1.80	1.04	83.2	1.44	0.91	0.88	0.55	0.55	0.63	0.62	0.72	9.0	8.7
C-10	M	53	1.74	1.39	88.2	1.36	0.79	0.82	0.57	0.54	0.58	0.60	1.03	9.0	8.9
C-11	F	76	1.55	0.93	87.1	1.38	0.86	0.88	0.50	0.52	0.63	0.63	0.68	7.0	7.2
C-12	F	63	1.64	1.02	91.7	1.31	0.78	0.69	0.58	0.58	0.58	0.55	0.78	7.6	7.4
C-13	F	67	1.59	1.22	96.8	1.24	0.74	0.70	0.51	0.53	0.59	0.57	0.99	7.7	7.7
C-14	M	48	1.73	1.37	95.2	1.26	0.76	0.76	0.49	0.51	0.61	0.60	1.09	9.1	9.0
C-15	F	42	1.63	1.01	81.2	1.48	0.91	0.92	0.58	0.55	0.61	0.63	0.68	6.9	6.8
C-16	F	60	1.60	1.01	100.0	1.20	0.78	0.78	0.42	0.43	0.65	0.65	0.85	6.7	6.5

**Table 4:** Neurological disorder subjects and extracted gait metrics. DLB, dementia with Lewy bodies; NPH, normal pressure hydrocephalus; VaD, vascular dementia; VaP, vascular Parkinsonism; MCI, mild cognitive impairment.

Subject ID	Gender	Age	Height (m)	Condition	Stride Length (m)	Cadence (steps/min)	Cycle Time (s)	L Stance Time (s)	R Stance Time (s)	L Swing Time (s)	R Swing Time (s)	L Stance Ratio	R Stance Ratio	Speed (m/s)	L Max. Clearance (cm)	R Max. Clearance (cm)	Turning Rate (degree/s)
1	M	72	1.74	Mixed	1.20	88.9	1.35	0.84	0.84	0.50	0.52	0.62	0.62	0.88	8.6	9.1	55.9
2	M	85	1.65	DLB	0.99	**109.3**	1.10	0.66	0.71	0.44	0.39	0.60	**0.65**	0.90	7.3	6.9	45.2
3	F	73	1.62	PD	1.11	99.9	1.20	0.76	0.70	0.45	0.49	**0.63**	0.58	0.92	**3.9**	5.3	50.3
4	F	82	1.58	PD	**0.50**	72.4	1.66	1.18	1.18	0.47	0.49	**0.72**	**0.71**	**0.30**	4.5	4.4	29.0
5	M	80	1.76	Mixed	1.10	**75.0**	1.60	0.87	1.02	0.71	0.60	0.55	0.63	**0.69**	7.7	8.4	**22.6**
6	M	88	1.80	PD	**0.36**	87.1	1.38	0.99	1.00	0.37	0.40	**0.73**	**0.72**	**0.26**	**2.1**	**3.7**	**19.3**
7	F	85	1.65	NPH	**0.81**	88.3	1.36	0.86	0.85	0.49	0.52	0.64	0.62	0.60	5.3	6.0	**28.8**
8	F	81	1.60	PD	**0.72**	**70.6**	1.70	1.15	1.12	0.55	0.58	**0.68**	**0.66**	**0.42**	5.2	5.9	**32.5**
9	F	80	1.60	Mixed	**0.73**	**82.6**	1.45	0.94	0.96	0.53	0.48	**0.64**	**0.67**	0.50	4.5	5.2	38.7
10	M	82	1.65	VaD	1.16	88.4	1.36	0.78	0.82	0.57	0.56	0.58	0.59	0.86	9.4	10.7	41.3
11	F	52	1.56	PD	**0.83**	**123.0**	0.98	0.57	0.67	0.40	0.32	0.59	**0.68**	0.85	**3.5**	**6.0**	59.1
12	F	90	1.60	PD	**0.53**	**119.3**	1.01	0.67	0.61	0.35	0.38	**0.66**	0.62	0.52	3.7	4.0	18.1
13	M	83	1.59	PD	0.93	95.6	1.26	0.77	0.76	0.48	0.50	0.62	0.60	0.74	6.9	6.1	45.1
14	M	76	1.82	MCI	1.31	92.2	1.30	0.76	0.76	0.56	0.53	0.58	0.59	1.01	9.0	9.3	45.1
15	M	84	1.69	VaP	**0.45**	90.9	1.32	0.89	0.87	0.43	0.45	**0.67**	**0.66**	**0.34**	**3.7**	**3.0**	**29.0**
16	F	82	1.50	DLB	0.83	83.8	1.43	0.96	0.84	0.47	0.59	**0.67**	0.59	0.58	5.2	4.8	35.8
17-ON	F	51	1.65	PD	0.82	98.6	1.22	0.85	0.88	0.37	0.33	**0.69**	**0.72**	0.67	**3.4**	**3.3**	30.2
17-OFF	F	51	1.65	PD	**0.55**	95.6	1.26	0.99	1.07	0.27	0.18	**0.79**	**0.85**	0.44	**2.9**	**2.4**	40.5
